# Influence of Tree Size and Application Rate on Expression of Thiamethoxam in Citrus and Its Efficacy Against *Diaphorina citri* (Hemiptera: Liviidae)

**DOI:** 10.1093/jee/toy001

**Published:** 2018-02-20

**Authors:** K W Langdon, R Schumann, L L Stelinski, M E Rogers

**Affiliations:** Department of Entomology and Nematology, Citrus Research and Education Center, University of Florida, Lake Alfred, FL, USA

**Keywords:** neonicotinoid, citrus, *Diaphorina citri*, soil application, thiamethoxam

## Abstract

Neonicotinoids are a key group of insecticides used to manage *Diaphorina citri* Kuwayama (Hemiptera: Liviidae), in Florida citrus. *Diaphorina citri* is the vector of *Candidatus* Liberibacter asiaticus, the presumed causal agent of huanglongbing, a worldwide disease of citrus. A two-season field study was conducted to evaluate the effect of tree size and application rate on the expression of thiamethoxam in young citrus following application to the soil. *D. citri* adult and nymph abundance was also correlated with thiamethoxam titer in leaves. Tree size and application rate each significantly affected thiamethoxam titer in leaf tissue. The highest mean thiamethoxam titer observed (33.39 ppm) in small trees (mean canopy volume = 0.08 m^3^) occurred after application of the high rate (0.74 g Platinum 75SG per tree) tested. There was a negative correlation between both nymph and adult abundance with increasing thiamethoxam titer in leaves. A concentration of 64.63 ppm thiamethoxam was required to reach a 1% probability of encountering a flush shoot with at least one adult *D. citri*, while 19.05 ppm was required for the same probability of encountering nymphs. The LC_90_ for the field population was 7.62 ppm thiamethoxam when administered through ingestion. Exposure to dosages as low as 7.62 ppm would likely result in sublethal exposure of some proportion of the population, which could exacerbate resistance development. Based on our results, subsequent work should investigate the use of neonicotinoids by foliar rather than soil application to maintain the chemical class in future insecticide management programs in Florida citrus.

Citrus (Rutaceae) is the largest agricultural commodity in Florida; approximately one-quarter million hectares valued at nearly 9.9 billion dollars were cultivated in 2015 (Hodges and Spreen 2015). This crop has come under severe decline in recent years due to the spread of the devastating citrus disease, huanglongbing (HLB). Huanglongbing is presumably the result of infection by the phloem-limited bacterium, *Candidatus* Liberibacter asiaticus (*C*Las), transmitted by the Asian citrus psyllid, *Diaphorina citri* Kuwayama (Hemiptera: Liviidae) (Halbert and Manjunath 2004, Bové 2006, Grafton-Cardwell et al. 2013). Following inoculation into a tree by a feeding *C*Las-infected psyllid, bacteria move from the infection site, through the vascular phloem to compromise the root system, which in turn deprives the tree canopy and reduces fruit yield (Halbert and Manjunath 2004, Bové 2006, Grafton-Cardwell et al. 2013). Huanglongbing was first detected in Florida in 2005 (Halbert 2005), just 7 yr after the discovery of *D. citri* (Halbert and Manjunath 2004). Vector management using insecticides quickly became the key strategy to reduce *D. citri* populations and consequent spread of HLB (Rogers 2008).

*D. citri* develop and reproduce rapidly, requiring as little as 15 d to complete the egg to adult life cycle under optimal environmental conditions (25–28°C) (Liu and Tsai 2000, Grafton-Cardwell et al. 2013). Adults typically seek volatile-emitting flush shoots as sites for oviposition (Patt and Sétamou 2010). Eggs hatch in 2–4 d, where newly emerged nymphs feed on phloem sap, thus acquiring *C*Las from the infected tree (Pelz-Stelinski et al. 2010). Because the probability of successful *C*Las acquisition by *D. citri* is higher for nymphs than for adults, the greatest risk of spread is from insects that acquire the bacteria during the nymph stage (Pelz-Stelinski et al. 2010). As nymphs become adults, they disperse and spread the bacteria to uninfected trees. An estimated 80–100% of *D. citri* found in Florida are *C*Las positive (Coy and Stelinski 2015); therefore, both nymphs developing on infected citrus tissue, as well as, infected adults feeding on new, uninfected plant material must be targets for successful vector suppression.

Young trees are defined as less than 8 ft in height that flush asynchronously and more often than mature trees (Hall and Albrigo 2007, Rogers 2012). Because young trees produce attractive flush shoots often throughout the year, they are at high risk of becoming infected with *C*Las throughout the year (Stansly and Rogers 2006). Insecticides have been important in mitigating *C*Las infection of young trees, especially before trees reach fruit-bearing age (Rogers 2012). Extension recommendations by the University of Florida suggest a rotation between soil-applied neonicotinoids and non-neonicotinoid foliar sprays to reduce *D. citri* populations in young tree groves (Rogers 2012, Rogers et al. 2014). Neonicotinoids are within the Insecticide Resistance Action Committee (IRAC) subgroup 4A, and act on the insect nicotinic acetylcholine receptor (nAChR) (IRAC 2017). Neonicotinoids are highly systemic and when applied to the soil, are taken up by the root system, and transported to the foliage via xylem channels (Elbert et al. 2008).

Three neonicotinoid insecticides are currently labeled for use in nonbearing citrus in Florida: thiamethoxam (Platinum 75 SG—Syngenta Crop Protection, Inc., Greensboro, NC), imidacloprid (Admire Pro 4.6F—Bayer CropScience, Research Triangle Park, NC), and clothianidin (Belay 2.13 SC—Valent USA Corporation, Walnut Creek, CA). Between 6 and 11 wk of *D. citri* control have been documented following the application of neonicotinoids to the soil (Qureshi and Stansly 2007, Qureshi and Stansly 2009, Ichinose et al. 2010, Sétamou et al. 2010, Rogers 2012). Residual activity of insecticides applied to the soil are likely influenced by factors such as soil type, application volume, irrigation, tree age and size, and environmental conditions such as rainfall. Moreover, neonicotinoid insecticides metabolize into various analytes, though the effect of any one resulting metabolite on *D. citri* mortality is unknown (Byrne et al. 2017). For example, thiamethoxam metabolizes into clothianidin (Nauen et al. 2003) and clothianidin further metabolizes into TZNG (*N*-(2-chlorothiazol-5-ylmethyl)-*N*-nitroguanidine) and TZMU (*N*-(2-chlorothiazol- 5-ylmethyl)-*N*-methylurea) (Kim et al. 2012). Nevertheless, to date, there has been scant information on movement, distribution, and persistence of soil-applied neonicotinoids or metabolites in citrus tissue in the field.

Previous insecticide trials evaluating soil-applied neonicotinoids used percent control relative to the untreated check or mean number of *D. citri* per sample size to assess efficacy (Qureshi and Stansly 2007, Qureshi and Stansly 2009, Ichinose et al. 2010, Sétamou et al. 2010, Byrne et al. 2012, Rogers 2012). Additional studies specifically quantified the concentration of neonicotinoids in leaf tissue following soil application using enzyme-linked immunosorbent assay (ELISA) (Castle et al. 2005, Garlapati 2009, Sétamou et al. 2010, Byrne et al. 2017). However, only one attempted to compare percent control with chemical titer within citrus leaf tissues. The lethal concentration of imidacloprid for *D. citri* was estimated between 200 and 250 parts per billion (ppb) by correlating percentage control of *D. citri* with imidacloprid titer (Sétamou et al. 2010). Because virtually all *D. citri* in Florida are assumed to be *C*Las positive, growers cannot tolerate any feeding on uninfected trees. In 2013, an estimated 1–3% of trees succumb to HLB infection annually in Florida groves, despite deliberate use of soil-applied neonicotinoids (Rogers 2013). Langdon and Rogers (2017) found that 62.19 ppm of imidacloprid was required to kill 90% of *D. citri* from a laboratory susceptible (LS) population through ingestion and hypothesized that the 200–250 ppb efficacy threshold set by Sétamou et al. (2010) may have been the result of sublethal feeding deterrence as opposed to lethal activity. Nevertheless, uneven uptake or distribution of neonicotinoids in citrus tissue may also result in exposure of *D. citri* to sublethal doses (Boina et al. 2009, Rogers 2012), which may aid in development of resistance to this particular chemistry. The problem of uneven distribution is compounded as trees grow, requiring increasing application rate. As rates are increased to match tree age and size, annual use limits become increasingly restricting for the development of effective year-long management strategies. Resistance to neonicotinoids was discovered in the field in 2009 (Tiwari et al. 2011a); however, reversion to susceptibility was reported by 2014 (Coy et al. 2016). This was attributed to increased rotations with foliar applied insecticides through area-wide spray programs. A more thorough understanding of the use of soil-applied neonicotinoids and factors that may stimulate the development of resistance is critical to maintaining effective use of this mode of action for management of HLB in Florida citrus.

The purpose of this study was to (i) quantify thiamethoxam expression over time within citrus tissue following soil application, (ii) evaluate efficacy of Platinum 75SG applied at two rates to young citrus trees of two sizes against *D. citri*, and (iii) quantify susceptibility of the *D. citri* field population to thiamethoxam by exposure through ingestion. By quantifying the temporal distribution of thiamethoxam in citrus leaf tissue with distinct tree sizes and application rates, we can more effectively refine management recommendations for the use of neonicotinoids in young citrus within the context of resistance management.

## Materials and Methods

### Insecticide Application and Citrus Leaf Sampling

A two-season field study was conducted during 2016 and 2017 to determine the concentration of thiamethoxam and resulting metabolites in citrus leaves over time following application of Platinum 75SG to the soil, based on application rate and tree size. We also determined the influence of thiamethoxam concentration on incidence and abundance of *D. citri* on treated trees over time. Untreated citrus (v. Hamlin/r.s. Swingle) trees of two nonbearing size classes were used in the study and defined as: large (mean canopy volume [MCV] approx. 1.34 m^3^) and small (MCV approx. 0.08 m^3^) in size. Large trees used in the study were field planted on 16-III-2015 and small trees were field planted on 01-VIII-2016. The same cohort of large and small trees was used during 2016 as used during 2017. Trees were planted to sandy soil comprising 96.8% sand, 1.6% silt, and 2% clay, with 1.04% organic matter and cation exchange capacity (CEC) of 6.7 meq/100g. Although rate calculations were based on the most common plant density of 140 trees per acre, due to space constraints, trees for this study were planted using a 2.4 m in-row spacing and 2.4 m between-row spacing, which provided sufficient separation to eliminate uptake of insecticides applied to an adjacent tree, confirmed by analysis of trees in the untreated control. The study was arranged in a randomized complete block design with six treatments and four replicates and each plot consisted of four trees. Prior to each insecticide application, tree canopy volume was measured, but no attempt was made to account for differences in individual tree size when identifying treatment plots. The first season insecticide application was made on 8-IX-2016 and the second season application was made on 20-I-2017. At the time of application, 237 ml of insecticide solution (deionized water + insecticide) was applied to the soil at the base of each tree trunk, which is the common application volume in the commercial setting. The high application rate was 0.74 g Platinum 75SG per tree (equiv. 3.67 oz wt product/ac on 140 trees/ac) and the low application rate was 0.37 g Platinum 75SG per tree (equiv. 1.83 oz wt product/ac on 140 trees/ac). Leaf tissue samples were collected prior to the application of insecticides and then weekly for 12 wk following the application. At each sample date, four mature, fully expanded leaves (~5–15 g) were randomly harvested from the outer canopy across each of the four trees within a plot. Leaves were placed into labeled paper bags and collectively stored by treatment in a plastic re-sealable bag at −20°C until residue analyses were conducted. Additionally, trees were evaluated weekly for the incidence and abundance of *D. citri* nymphs and adults. At each sample date, the number of *D. citri* nymphs and adults across 10 flush terminals per plot, and the number of nymph and adult infested terminals within each plot were counted and reported separately.

### Extraction and Leaf Tissue Analysis

Leaf material from each plot was ground to fine powder using liquid nitrogen with mortar and pestle. A ~5 g subsample of leaf powder was weighed and transferred to a 20 ml glass vial with a Teflon-lined cap and stored at −20°C until extraction; the exact weight of each sample was recorded for conversion of analyte concentration to fresh leaf weight. Extraction was conducted with QuEChERS (Anastassiades et al. 2003) in 15 ml acetonitrile using preweighed reagent sachets (United Chemical Technologies, #ECQUEU7-MP). A cleanup step was then conducted in which chlorophyll was removed from the acetonitrile extract using ChloroFiltr polymeric based sorbent tubes (United Chemical Technologies, Horsham, PA, # ECMPSGG15CT). The supernatant from cleanup was filtered through a 20 µm Teflon filter into an auto sampler vial. Separation and quantification of analytes was accomplished using Ultra-High Performance Liquid Chromatography with a C-18 column coupled to a Thermo TSQ Quantum mass spectrometer (UHPLC-MS). The LOQ was 5 ng/g for imidacloprid, 10 ng/g for clothianidin, thiamethoxam, and 5-OH, and 25 ng/g for olefin, TZMU, and TZNG. The LOD was 1.5 ng/g for imidacloprid, 3.2 ng/g for clothianidin and thiamethoxam, 3.0 ng/g for 5-OH, 8.0 ng/g for olefin, and 8.3 ng/g for TZMU and TZNG. The aqueous mobile phase was 0.1% formic acid in water and the polar modifying phase was 0.1% formic acid in acetonitrile. Samples were run against standards to construct a five-point linear curve in a concentration range of 0.5–50 ppm, and then against a five-point standard curve in the range of 5–300 ppb. The concentration represented by the curve (in extract solution) was then converted back to micrograms/gram leaf tissue using the exact sample weight. The standards were matrix matched to compensate for signal suppression effects of the matrix. Plant tissue free of all four analytes was extracted using QuEChERS as outlined above in order to obtain a blank matrix for mixing working standards. Primary standards were made using technical grade material (97.6–99.9%) in acetonitrile; technical grade material was obtained from Syngenta Crop Protection, Inc., Greensboro, NC, or Valent USA Corporation, Walnut Creek, CA). A set of working standards encompassing the linear range of concentrations was prepared from the primary standards, again in acetonitrile. To prepare a range of working standards in blank plant matrix, a 1,000 µl aliquot of blank plant matrix was dry evaporated under nitrogen. The residue was then reconstituted with a 1,000 µl aliquot of standard acetonitrile. The solution was sonicated to ensure a homogeneous product and passed through a 0.2 µm PTFE filter prior to injection, as were unknowns.

### Insect Biological Assays

#### Lab Culture

The LS strain was reared in continuous culture at the University of Florida Citrus Research and Education Center in Lake Alfred on *Murraya koenigii* maintained at 27°C with RH 65% with a photoperiod of 14:10 (L:D) h. The colony did not receive any exposure to insecticides following establishment in 2005 and routine quantitative real time (qPCR) testing as described in Pelz-Stelinski et al. (2010) was used to confirm the colony was *C*Las-free. Adult *D. citri* were aspirated from host rearing plants and used in laboratory bioassays during the same day to reduce unintended mortality.

#### Field Collection of *D. citri*

*D. citri* adults were collected prior to the first field application to establish baseline susceptibility of the field population to thiamethoxam in the laboratory. Adults were aspirated from citrus foliage in the field and transported to the lab within labeled plastic aspirator vials. Laboratory assays were conducted during the same day that *D. citri* were collected from the field to reduce unintended mortality.

#### Ingestion Bioassay

The ingestion assay was comprehensively described in Langdon and Rogers (2017). Briefly, a 30% sucrose solution was used as the base artificial diet. Serial dilution was conducted to form eight doses of spiked diet using formulated Platinum 75SG (750 g thiamethoxam kg, Syngenta Crop Protection, Greensboro, NC). The caps of 5 ml snap-cap centrifuge tubes (Eppendorf Tubes, Hamburg, Germany, Cat. No.: 0030119401) were filled with 0.7 ml of each prepared dose. Parafilm M (Bemis, Neenah, WI, Cat. No.: PM-992) was stretched over each diet-filled cap. Four to six adult *D. citri* were loaded into each centrifuge tube and the diet-filled cap was reinstalled. Tubes were held upright in a tube tray at 27°C, 70% relative humidity, with a 14:10 (L:D) h photoperiod for 72 h. One replicate was defined as a single tube and 10 replicates were used for each dose. Between 40 and 60 adults were tested for each dose. After 72 h, insects were scored as alive (full function), moribund (insects lacking coordinated movement), or dead (no movement upon disturbing). Moribund insects were classified as dead for data analysis.

#### Statistical Analyses

Chemical titer data were averaged over replicate and subjected to a general linear mixed model using SASv9.4 (Proc GLIMMIX, SAS Institute 2013) to test for year by treatment interactions. Means were square root transformed prior to analysis to achieve homogeneity of variance meeting the assumptions of the model, as checked by visual examination of the residuals to ensure constant variance and normality. Additionally, chemical titer data were averaged over replicate and year and subjected to a general linear mixed model using SASv9.4 (Proc GLIMMIX, SAS Institute 2013) to test for tree size by application rate interactions. Means were square root transformed prior to analysis to achieve homogeneity of variance and meet the assumptions of the model. For tests of differences between treatments, data were subjected to a nonparametric multiple comparisons test where mean separations indicate differences between treatments within the same sample week at α ≤ 0.05.

Insect count data were in monotonic distribution; therefore, data were subjected to Spearmans rank-order correlation using JMP (JMP Version 13, SAS Institute 2007) to determine if concentration of thiamethoxam, or the metabolites clothianidin, TZNG, or TZMU influenced *D. citri* incidence on leaves. Correlations were estimated using the Restricted Maximum Likelihood (REML) method. Additionally, nymph and adult incidence data were subjected to Probit analysis using SAS v9.4 (Proc Probit, SAS Institute 2013) to determine the probability of encountering a flush terminal containing at least one nymph or one adult based on thiamethoxam titer.

## Results

### Chemical Titer in Leaf Tissue

No year by treatment interaction was observed for thiamethoxam (*F*_1, 5_ = 0.9982; *P* = 0.4328), clothianidin (*F*_1, 5_ = 1.4132; *P* = 0.2429), or TZMU (*F*_1, 5_ = 2.1454; *P* = 0.0822); however, a year by treatment interaction was observed for TZNG (*F*_1, 5_ = 3.5969; *P* = 0.0097). For TZNG, a larger magnitude of difference was observed in 2016 compared with 2017; however, the order of the treatment effects was the same across years. Therefore, data were combined across years for each of the four chemicals to evaluate the effect of treatment on chemical titer.

A tree size by application rate interaction was observed for thiamethoxam (F_1, 27_ = 15.11; *P* = 0.0006), clothianidin (*F*_1, 27_ = 11.66; *P* = 0.0020), and TZMU (*F*_1, 27_ = 43.60; *P* < 0.0001); however, no tree size by application rate interaction was observed for TZNG (*F*_1, 27_ = 0.8936; *P* = 0.3529). Main effects of tree size and application rate were therefore analyzed separately. Tree size influenced thiamethoxam titer (*F*_1, 27_ = 180.5; *P* < 0.0001), where higher thiamethoxam titers were expressed in small trees than in large trees. Tree size also influenced titer of clothianidin (*F*_1, 27_ = 187.2; *P* < 0.0001), TZMU (*F*_1, 27_ = 233.6; *P* < 0.0001), and TZNG (*F*_1, 27_ = 263.3; *P* < 0.0001). Additionally, the rate of application affected thiamethoxam titer (*F*_1, 27_ = 44.24; *P* < 0.0001), where the application of the high rate resulted in a significantly more thiamethoxam in leaf tissue than at the low rate. Application rate also affected clothianidin (*F*_1, 27_ = 39.35; *P* < 0.0001), TZMU (*F*_1, 27_ = 58.02; *P* < 0.0001), and TZNG (*F*_1, 27_ = 27.03; *P* < 0.0001) by increasing measured titer with increased application rate.

The high rate (0.74 g Platinum 75SG per tree) applied to the small tree size resulted in the highest thiamethoxam titer observed during each week following application; the peak mean concentration occurred 2 and 3 wk following the application of 33.39 and 33.29 ppm of thiamethoxam, respectively (Table 1). Mean thiamethoxam titer peaked at 12.53 ppm by 3 wk after the application of the low rate application (0.37g) of Platinum 75SG per small tree; however, no significant difference in titer was observed between rates in small trees 3 wk following application (*χ*^2^ = 36.53, *P* < 0.0001, Table 1). Peak thiamethoxam titer was observed at 2 (2.86 ppm) and 4 (2.47 ppm) wk after the high rate (0.74 g Platinum 75SG per tree) application to large trees, and at 2 wk (0.69 ppm) following the low rate (0.37 g Platinum 75SG per tree) application to large trees. At 2 and 4 wk after application, significantly more thiamethoxam was found after the high rate than the low rate application to large trees (*χ*^2^ = 40.27, *P* < 0.0001 and *χ*^2^ = 43.91, *P* < 0.0001, respectively, Table 1).

**Table 1.** Mean parts per million (ppm) of thiamethoxam (95% CI) found in citrus leaf tissue

Tree size	Application rate per tree	Weeks after application												
		0	1	2	3	4	5	6	7	8	9	10	11	12
Small 0.08 m^3^ MCV^*a*^	Untreated	0	0	0	0	0	0	0	0	0	0	0	0	0
	0.37 g Platinum 75SG	0	7.53bc (3.46–11.61)	6.92b (3.20–10.63)	12.53b (8.27–16.79)	7.51c (3.56–11.45)	7.03b (4.29–9.77)	3.27b (2.14–4.39)	2.39c (1.03–3.76)	1.42b (0.95–1.88)	1.14b (0.72–1.56)	0.56c (0.30–0.82)	0.37b (0.24–0.49)	0.30bc (0.13–0.46)
	0.74 g Platinum 75SG	0	13.58c (6.07–21.09)	33.39c (16.54–50.23)	33.29b (17.20–49.39)	26.90d (15.37–38.44)	28.10c (17.01–39.19)	16.29c (9.08–23.51)	11.03d (6.18–15.87)	6.09c (2.96–9.23)	3.96c (2.09–5.83)	2.90d (1.48–4.33)	1.52c (0.48–2.57)	0.80c (0.43–1.15)
Large 1.34 m^3^ MCV^a^	Untreated	0	0	0	0	0	0	0	0	0	0	0	0	0
	0.37 g Platinum 75SG	0	0.61ab (0.00–3.14)	0.69a (0.12–1.27)	0.53a (0.00–2.03)	0.39a (0.18–0.60)	0.17a (0.00–2.03)	0.17a (0.00–0.89)	0.16ab (0.04–0.27)	0.11a (0.00–0.58)	0.09a (0.00–0.31)	0.03ab (0.00–0.06)	0.03a (0.00–0.11)	0.05ab (0.00–0.10)
	0.74 g Platinum 75SG	0	1.59abc (0.00–4.58)	2.86b (1.00–4.72)	1.90a (0.63–3.17)	2.47bc (0.00–5.70)	0.67a (0.00–2.44)	0.70a (0.01–1.39)	0.39b (0.20–0.59)	0.30a (0.00–0.80)	0.28ab (0.03–0.53)	0.19bc (0.08–0.30)	0.14ab (0.04–0.25)	0.08ab (0.03–0.14)
	*P*-value	–	<0.0001	<0.0001	<0.0001	<0.0001	<0.0001	<0.0001	<0.0001	<0.0001	<0.0001	<0.0001	<0.0001	<0.0001
	*χ* ^2^	–	28.95	40.27	36.53	43.91	35.18	35.65	40.89	35.23	34.89	41.27	35.08	32.74

Mean separations within columns indicate differences between treatments within weekly samples. Values sharing the same letter do not differ significantly at α ≤ 0.05.

^*a*^Mean volume of citrus tree canopy.

The thiamethoxam metabolite, clothianidin, peaked at 5 wk post application of both the high rate (15.44 ppm) and the low rate (6.29 ppm) of Platinum 75SG in trees of small size (Table 2). Following application to the large size trees, clothianidin titer peaked at 4 wk with both the high rate (1.69 ppm) and the low rate (0.48 ppm). Very low levels (<2 ppm) of the clothianidin metabolites, TZMU (Table 3) and TZNG (Table 4) were detected in leaf tissues from trees following the application of Platinum 75SG to the soil.

**Table 2.** Mean parts per million (ppm) of clothianidin (95% CI) found in citrus leaf tissue

Tree size	Application rate per tree	Weeks after application												
		0	1	2	3	4	5	6	7	8	9	10	11	12
Small 0.08 m^3^ MCV^*a*^	Untreated	0	0	0	0	0	0	0	0	0	0	0	0	0
	0.37 g Platinum 75SG	0	1.99b (0.77–3.21)	2.45cd (0.81–4.09)	5.82c (4.07–7.56)	4.50cd (2.85–6.15)	6.29b (5.07–7.52)	4.33b (2.45–6.21)	2.65b (1.57–3.73)	1.84b (1.39–2.29)	1.46b (0.94–1.98)	0.85b (0.52–1.17)	0.55b (0.43–0.66)	0.40b (0.22–0.58)
	0.74 g Platinum 75SG	0	3.27b (1.46–5.08)	8.62d (4.27–12.96)	12.12c (6.77–17.47)	13.09d (8.34–17.85)	15.44b (7.32–23.57)	14.02c (7.95–20.09)	11.53c (7.67–15.39)	7.25c (3.63–10.88)	5.01c (3.00–7.02)	5.02c (3.04–6.99)	2.21c (1.05–3.37)	1.45c (0.84–2.05)
Large 1.34 m^3^ MCV^*a*^	Untreated	0	0	0	0	0	0	0	0	0	0	0	0	0
	0.37 g Platinum 75SG	0	0.26ab (0.00–0.97)	0.39b (0.00–0.82)	0.38b (0.17–0.58)	0.48b (0.10–0.87)	0.21a (0.00–0.78)	0.19a (0.00–0.81)	0.18a (0.00–0.66)	0.14a (0.00–0.81)	0.09a (0.00–0.56)	0.04a (0.00–0.38)	0.03a (0.00–0.22)	0.05a (0.00–0.14)
	0.74 g Platinum 75SG	0	0.68ab (0.00–1.42)	1.15bc (0.45–1.85)	1.06b (0.38–1.73)	1.69bc (0.00–3.75)	0.86a (0.37–1.36)	0.74a (0.26–1.23)	0.40a (0.00–0.90)	0.33a (0.00–0.98)	0.30ab (0.00–0.77)	0.29ab (0.00–0.60)	0.17a (0.00–0.36)	0.09a (0.00–0.18)
	*P*-value	–	<0.0001	<0.0001	<0.0001	<0.0001	<0.0001	<0.0001	<0.0001	<0.0001	<0.0001	<0.0001	<0.0001	<0.0001
	χ^2^	–	29.17	40.86	42.45	43.16	32.66	36.60	35.43	35.16	32.25	32.20	34.93	35.47

Mean separations within columns indicate differences between treatments within weekly samples. Values sharing the same letter do not differ significantly at α ≤ 0.05.

^*a*^Mean volume of citrus tree canopy.

**Table 3.** Mean parts per million (ppm) of TZMU (95% CI) found in citrus leaf tissue

Tree size	Application rate per tree	Weeks after application												
		0	1	2	3	4	5	6	7	8	9	10	11	12
Small 0.08 m^3^ MCV^*a*^	Untreated	0	0	0	0	0	0	0	0	0	0	0	0	0
	0.37 g Platinum 75SG	0	0.04a (0.02–0.06)	0.05a (0.01–0.08)	0.11b (0.04–0.17)	0.09b (0.05–0.13)	0.05a (0.00–0.09)	0.07ab (0.02–0.12)	0.01a (0.00–0.03)	0.01a (0.00–0.03)	0.01ab (0.00–0.02)	0a	0a	0a
	0.74 g Platinum 75SG	0	0.08a (0.02–0.15)	0.44b (0.22–0.65)	0.38c (0.20–0.57)	0.52c (0.32–0.72)	0.40b (0.25–0.54)	0.38b (0.19–0.57)	0.23b (0.10–0.36)	0.15b (0.09–0.22)	0.10b (0.05–0.15)	0.06a (0.00–0.12)	0.02a (0.00–0.04)	0.01a (0.00–0.02)
Large 1.34 m^3^ MCV^*a*^	Untreated	0	0	0	0	0	0	0	0	0	0	0	0	0
	0.37 g Platinum 75SG	0	0a	0a	0a	0a	0a	0a	0a	0a	0a	0a	0a	0a
	0.74 g Platinum 75SG	0	0a	0a	0a	0.01ab (0.00–0.04)	0.01a (0.00–0.01)	0a	0a	0a	0a	0a	0a	0a
	*P*-value	-	0.0008	<0.0001	<0.0001	<0.0001	0.0005	<0.0001	0.0014	<0.0001	0.0030	0.0911	0.0174	0.6178
	χ^2^	-	21.13	27.40	33.82	39.89	22.11	26.59	19.80	25.37	17.93	9.49	13.73	3.54

Mean separations within columns indicate differences between treatments within weekly samples. Values sharing the same letter do not differ significantly at α ≤ 0.05.

^*a*^Mean volume of citrus tree canopy.

**Table 4.** Mean parts per million (ppm) of TZNG (95% CI) found in citrus leaf tissue

Tree size	Application rate per tree	Weeks after application												
		0	1	2	3	4	5	6	7	8	9	10	11	12
Small 0.08 m^3^ MCV^*a*^	Untreated	0	0	0	0	0	0	0	0	0	0	0	0	0
	0.37 g Platinum 75SG	0	0.17ab (0.07–0.27)	0.25bc (0.13–0.37)	0.68c (0.50–0.87)	0.66c (0.51–0.81)	0.65b (0.46–0.83)	0.87c (0.53–1.21)	0.69c (0.57–0.80)	0.59b (0.45–0.74)	0.57b (0.42–0.72)	0.39b (0.32–0.47)	0.33b (0.24–0.41)	0.22b (0.15–0.29)
	0.74g Platinum 75SG	0	0.24b (0.12–0.36)	0.41c (0.28–0.55)	0.77c (0.60–0.95)	0.70c (0.51–0.89)	1.39c (0.92–1.86)	1.36c (0.87–1.86)	1.27c (0.78–1.75)	1.19b (0.66–1.73)	0.81b (0.40–1.23)	0.89c (0.68–1.10)	0.66b (0.41–0.90)	0.52c (0.42–0.63)
Large 1.34 m^3^ MCV^*a*^	Untreated	0	0a	0a	0a	0a	0a	0a	0a	0a	0a	0a	0a	0a
	0.37g Platinum 75SG	0	0.02ab (0.00–0.08)	0.08ab (0.00–0.18)	0.12b (0.02–0.23)	0.15ab (0.00–0.30)	0.04a (0.00–0.13)	0.09b (0.05–0.13)	0.06ab (0.01–0.11)	0.04a (0.00–0.12)	0.02a (0.00–0.10)	0.01a (0.00–0.05)	0.01a (0.00–0.04)	0.01a (0.00–0.03)
	0.74g Platinum 75SG	0	0.06ab (0.00–0.13)	0.12ab (0.01–0.22)	0.20b (0.06–0.35)	0.33bc (0.10–0.56)	0.16a (0.05–0.28)	0.19b (0.08–0.30)	0.13b (0.07–0.19)	0.12a (0.04–0.20)	0.11a (0.03–0.19)	0.09a (0.04–0.14)	0.04a (0.00–0.10)	0.03a (0.00–0.06)
	*P*-value	–	0.0004	<0.0001	<0.0001	<0.0001	<0.0001	<0.0001	<0.0001	<0.0001	<0.0001	<0.0001	<0.0001	<0.0001
	X^2^	–	22.64	30.73	41.08	37.89	38.01	40.59	40.03	34.70	35.01	37.49	34.14	34.95

Mean separations within columns indicate differences between treatments within weekly samples. Values sharing the same letter do not differ significantly at α ≤ 0.05.

^*a*^Mean volume of citrus tree canopy.

### Baseline Susceptibility of Field *D*. *citri* Population

The lethal concentration required to kill half of the field-collected (Vero Beach) population (LC_50_) by ingestion was 0.20 ppm of thiamethoxam, while the LC_50_ was 0.11 ppm for the lab susceptible population (Table 5). A comparison of these results indicates that the field population investigated during this experiment was fully susceptible to thiamethoxam, exhibiting a resistance ratio (RR_50_) of 1.82. Furthermore, the lethal concentration required to kill 99% of the field-collected population by ingestion (LC_99_) was 147.91 ppm thiamethoxam.

**Table 5.** Response of LS and field-collected *D. citri* to thiamethoxam administered by ingestion

Strain	N^*a*^	Slope + SE	X^2^	LC_50_^*b*^	95% CL	LC_90_^*b*^	95% CL	LC_99_^*b*^	95% CL	RR_50_^*c*^
Vero Beach	356	0.35 + 0.04	84.53	0.20	(0.10–0.34)	7.62	(4.10–17.73)	147.91	(52.34–693.09)	1.82
Lab Susceptible	404	0.34 + 0.04	73.58	0.11	(0.05–0.21)	4.94	(2.63–11.75)	106.45	(36.17–555.23)	–

Shaded area represents data from Langdon and Rogers (2017).

^*a*^Number of adult *D. citri* tested.

^*b*^Parts per million (ppm) of active ingredient.

^*c*^Ratio of Vero Beach LC_50_ divided by Lab Susceptible LC_50_.

### Relationship Between *D*. *citri* Incidence and Chemical Titer

**Fig. 1.**
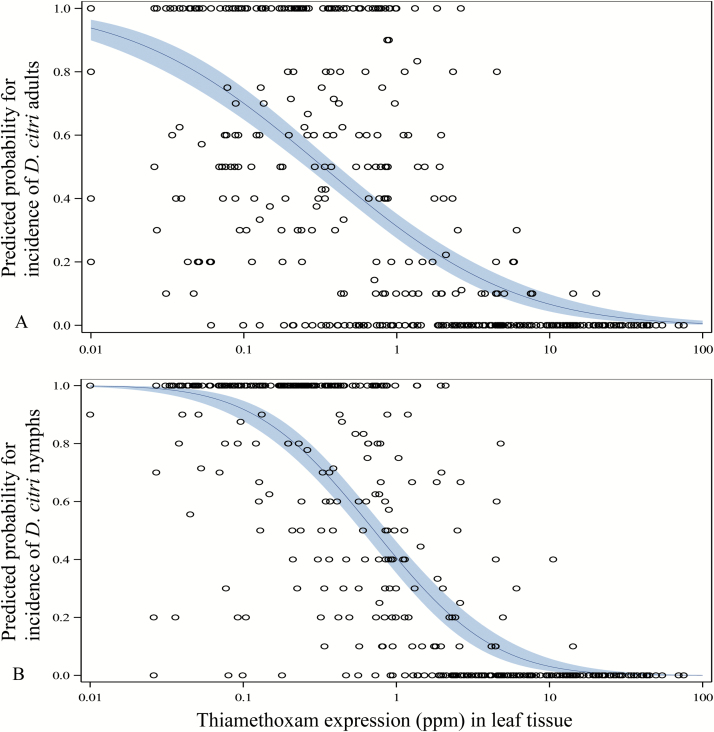
Predicted probability for the incidence of insects based on thiamethoxam titer. (A) Incidence of *D. citri* adults. (B) Incidence of *D. citri* nymphs.

Adult incidence was negatively correlated with increasing thiamethoxam (Spearman’s *ρ* = −0.6440; Table 6), clothianidin (Spearman’s *ρ* = −0.6320; Table 6), TZMU (Spearman’s *ρ* = −0.5429; Table 6), and TZNG (Spearman’s *ρ* = −0.6117; Table 6) titer. Likewise, nymph incidence was negatively correlated with increasing thiamethoxam (Spearman’s *ρ* = −0.7010; Table 6), clothianidin (Spearman’s *ρ* = −0.6913; Table 6), TZMU (Spearman’s *ρ* = −0.6051; Table 6), and TZNG (Spearman’s *ρ* = −0.6655; Table 6) titer. An estimated 64.63 ppm (95% CL: 34.40–147.16) of thiamethoxam was required to achieve a 1% probability of encountering a flush terminal with one *D. citri* adult (Fig. 1A). Additionally, a thiamethoxam titer of 0.329 ppm (329 ppb) yielded a 50% probability of encountering a flush terminal with one *D. citri* adult (Supp Table 1 [online only]). In contrast, an estimated 19.05 ppm of thiamethoxam was required to achieve a 1% probability of encountering a flush terminal with one *D. citri* nymph (Fig. 1B); 0.715 ppm of thiamethoxam (715 ppb) yielded a 50% probability of encountering a flush terminal with one *D. citri* nymph (Supp Table 2 [online only]).

**Table 6.** Nonparametric Spearman correlation between mean number of adult or nymph *D. citri* per terminal an chemical titer during 2016 and 2017 field seasons

Variable	by Analyte	Spearman ρ	*P*-value
Mean number of *D. citri* adults per terminal (*n* = 10 terminals)	thiamethoxam	−0.6440	<0.0001
	clothianidin	−0.6320	<0.0001
	TZMU	−0.5429	<0.0001
	TZNG	−0.6117	<0.0001
Mean number of *D. citri* nymphs per terminal (*n* = 10 terminals)	thiamethoxam	−0.7010	<0.0001
	clothianidin	−0.6913	<0.0001
	TZMU	−0.6051	<0.0001
	TZNG	−0.6655	<0.0001

**Fig. 2.**
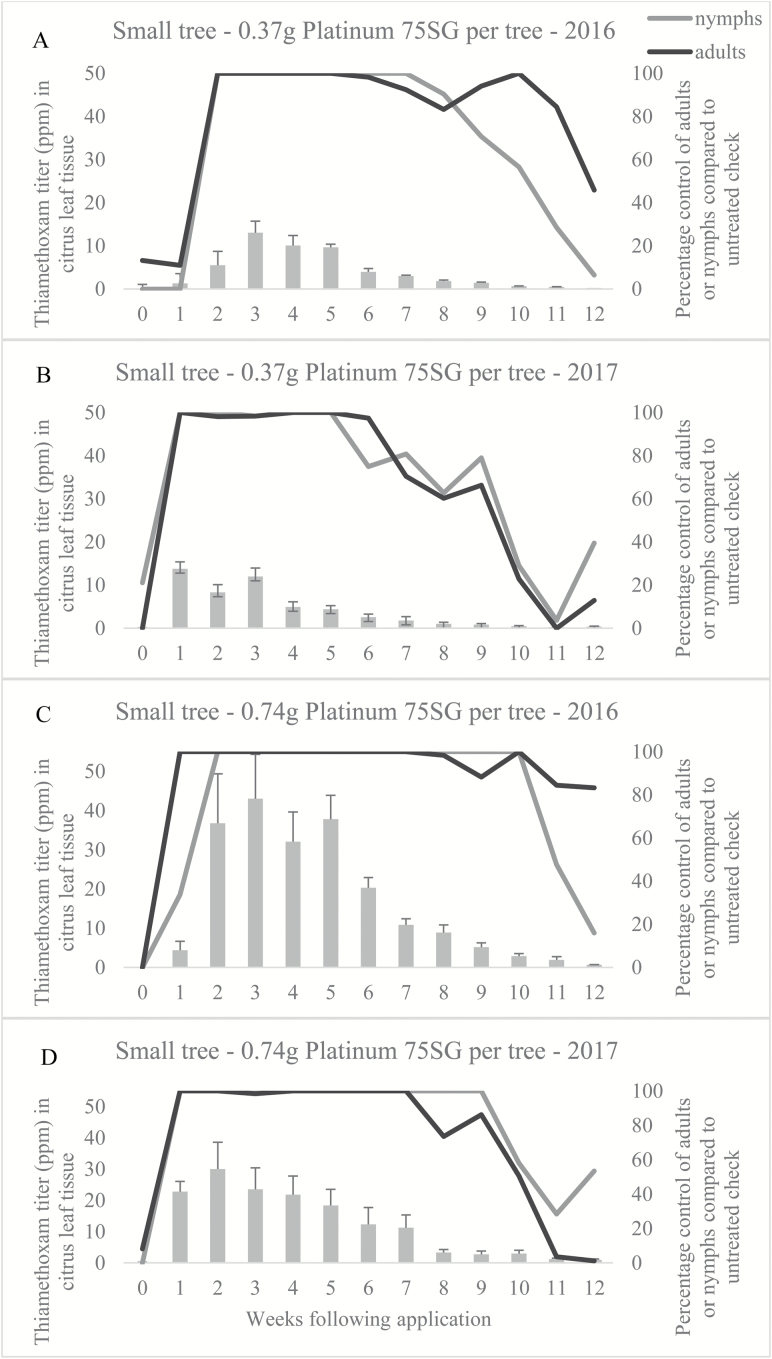
Comparison of thiamethoxam titer and percentage insect control in trees of the small size during 2016 and 2017 field seasons. (A) Low rate applied to small trees in 2016. (B) Low rate applied to small trees in 2017. (C) High rate applied to small trees in 2016. (D) High rate applied to small trees in 2017.

**Fig. 3.**
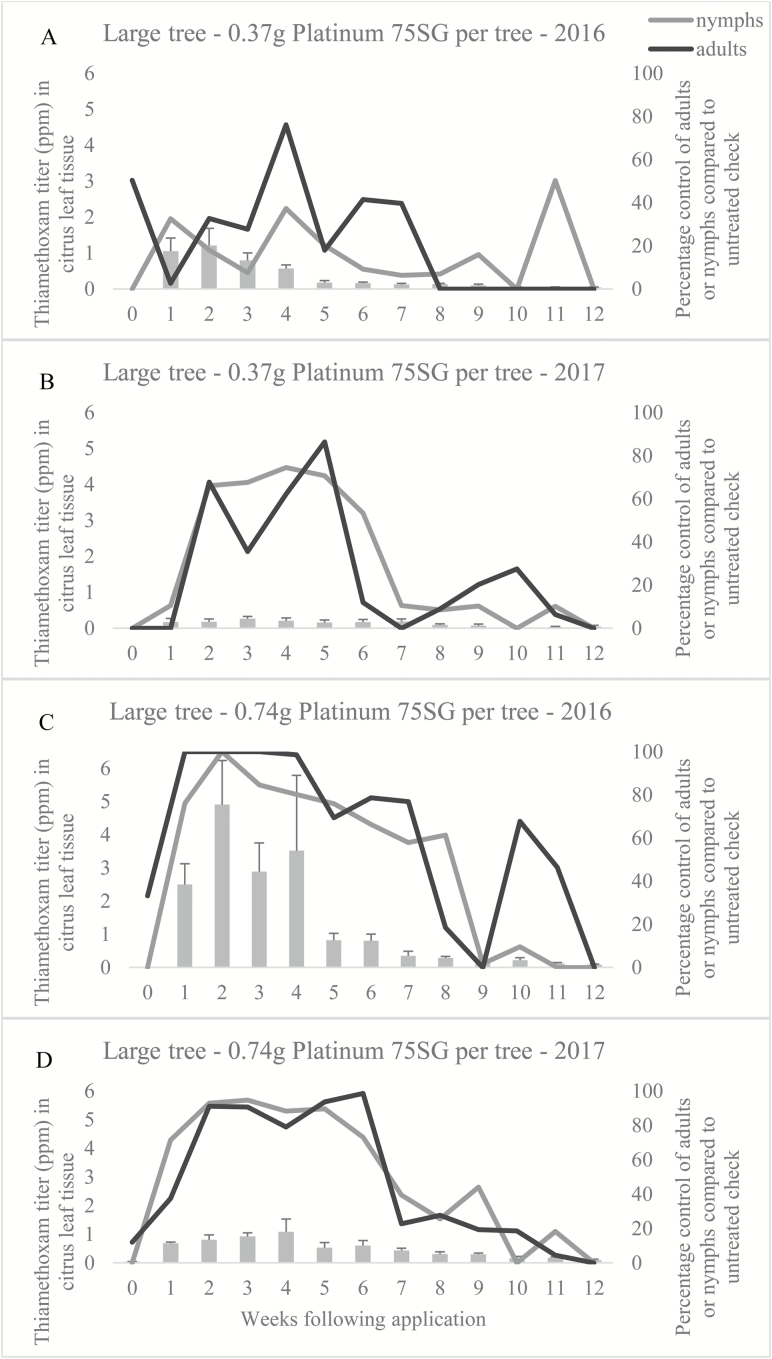
Comparison of thiamethoxam titer and percentage insect control in trees of the large size during 2016 and 2017 field seasons. (A) Low rate applied to large trees in 2016. (B) Low rate applied to large trees in 2017. (C) High rate applied to large trees in 2016. (D) High rate applied to large trees in 2017.

Approximately 5 wk of nymph and adult control was observed in 2016 and 2017 (Fig. 2A and B, respectively), following application of the low rate of Platinum 75SG to the soil beneath small trees, where ‘control’ is defined as 100% compared with the untreated check. In contrast, 10 and 9 wk of nymph and 8 and 7 wk of adult control was observed in 2016 (Fig. 2C) and 2017 (Fig. 2D), respectively, following the high rate application to trees of the small size. The low rate applied to the large tree size did not offer complete control of nymphs or adults at any time during 2016 or 2017 (Fig. 3A and B, respectively). During 2016, complete adult control was observed only during weeks 1 through 3 following the high rate application to trees of the large size (Fig. 3C), but the same level of adult control was not observed during 2017 (Fig. 3D); nymph control reached 100% only at 2 wk after application in 2016 (Fig. 3C).

## Discussion

The goals of this study were to: i) quantify uptake and expression of thiamethoxam in young citrus trees of two sizes following the application of Platinum 75SG to the soil at two rates; ii) evaluate residual efficacy against *D. citri* nymphs and adults; and iii) quantify the susceptibility of the field population of *D. citri* to thiamethoxam by comparing lethal concentration values with vector control as determined by population monitoring. This was the first formal investigation to use UHPLC-MS for quantification of the four analytes of Platinum 75SG (thiamethoxam, clothianidin, TZNG, and TZMU) in citrus leaf tissues following soil application in the field. Thiamethoxam is a neonicotinoid precursor to clothianidin (Nauen et al. 2003) and clothianidin is known to metabolize into TZNG and TZMU (Kim et al. 2012). While clothianidin is effective against *D. citri*, little is known about the influence of specific dosage on *D. citri* feeding behavior or mortality (Byrne et al. 2017). Langdon and Rogers (2017) found that the LC_50_ of thiamethoxam and clothianidin to the LS population by ingestion was 0.11 and 0.09 ppm, respectively, while the LC_90_ was 4.94 and 9.35 ppm, respectively. Because a higher dose of clothianidin was required to achieve the same level of mortality at the 90% lethal concentration, it is unlikely that mortality observed in the current study is the result of exposure to the metabolite, clothianidin alone. Furthermore, the concentration of each of the three metabolites is directly dependent upon the concentration of thiamethoxam. Consequently, we cannot determine how any one metabolite, including clothianidin, affects *D. citri* mortality in this study. While understanding the role of each metabolite is beyond the scope of this investigation, it is possible that the combination of multiple metabolites has an additive effect on *D. citri* mortality as suggested by Byrne et al. (2017). Byrne et al. (2017) demonstrated a strong correlation between thiamethoxam and the metabolite, clothianidin, but were unable to determine whether mortality was the result of either thiamethoxam or clothianidin, or a combination of the two. Although earlier studies used ELISA to quantify expression of neonicotinoids (Castle et al. 2005, Garlapati 2009, Sétamou et al. 2010), they did not quantify metabolites of thiamethoxam nor manipulate tree size or application rate to study effects on thiamethoxam titer.

The target concentration threshold of imidacloprid following application to the soil was 200–250 ppb based on the report by Sétamou et al. (2010). Since a correlation between *D. citri* abundance and clothianidin or thiamethoxam titer did not exist, 200–250 ppb became the assumed efficacy threshold concentration for all neonicotinoids (K.W.L., personal observations). In the current investigation, peak levels of nearly 3 ppm (3000 ppb) and 0.7 ppm (700 ppb) of thiamethoxam were measured when the respective high and low rates tested were applied to trees of the large size. When the high and low rates were applied to trees of the small size, peak concentrations were nearly 18- and 11-fold higher, respectively, than when applied to the large tree size. Each titer was well above the 250 ppb upper threshold set for imidacloprid, suggesting that efficacy should be expected in both tree sizes at the rates tested. However, despite the high titers observed in our study, we failed to reach a mean dose high enough to provide a 95% confidence (34.40 ppm) of only a 1% probability of encountering a flush shoot with at least one adult *D. citri*. We did, however, observe a mean concentration high enough in only the small tree size at both rates, to achieve a 95% confidence (12.17 ppm) of only a 1% probability of encountering a flush shoot with at least one nymph, albeit that titer was reached only during the third week of the investigation in the low rate treatment; the high rate treatment exceeded 12.17 ppm between weeks 1 and 6.

The LC_90_ for the field population was 7.62 ppm. This was exceeded during the first 3 wk after the low rate was applied to trees of the small size and was exceeded for the first 7 wk after the high rate was applied to trees of the small size. Given that nearly 100% control of adults was observed for up to 8 wk following application of either rate to the small tree size, it is possible that dosages as low as 7.62 ppm are capable of deterring some proportion of *D. citri* from feeding and, therefore, may offer nonlethal control of *D. citri* in young citrus trees. Electropenetrography was used to demonstrate that imidacloprid applied to the soil in the greenhouse can reduce phloem feeding by *D. citri*; however, phloem feeding was not prevented in either study (Serikawa et al. 2012, Miranda et al. 2016). The rate of imidacloprid applied to the soil in each study was known, but the imidacloprid titer within the phloem sap was unknown. Langdon and Rogers (2017) defined insecticide-mediated feeding deterrence of *D. citri* as ‘gustatory avoidance of less or nonsuitable feeding sources.’ As adults search for a suitable host plant and are exposed to citrus tissue containing more than 7.62 ppm of thiamethoxam, adults may move to find a leaf containing a lower concentration within the same plant or move to a new host plant that contains a concentration sufficiently low to be suitable for feeding. This movement away from the treated foliage or tree may result in a perception of control upon visual assessment, but should not be confused with mortality. Survival of exposed insects may promote the development of resistance to thiamethoxam and likely other chemistries within the same mode of action in populations of *D. citri*. Thiamethoxam is known to metabolize into clothianidin, which acts on the same receptor site as imidacloprid (Nauen et al. 2003), supporting a high likelihood of cross-resistance. Imidacloprid resistant *D. citri* express higher levels of detoxifying enzymes, including general esterase, glutathione *S*-transferase, and cytochrome P_450_ monooxygenases (Tiwari et al. 2011a). Reduced penetration, target-site insensitivity, and mutations in detoxifying enzymes may also play a role in resistance (Tiwari et al. 2011a). Tiwari et al. (2011b) found five family 4 cytochrome P_450_ genes induced by imidacloprid exposure. Imidacloprid and thiamethoxam are within the same chemical subgroup (4A); therefore, cross-resistance between the two chemistries is of concern. Additionally, thiamethoxam persisted at very low levels (~0.05–0.80 ppm) during the final evaluation of this investigation (12 wk following application). The duration at which sublethal doses in this range will persist is unknown; if doses in this range do not inhibit feeding activity, it may further increase the likelihood and rate of resistance development.

*D. citri* resistance to neonicotinoids has been recently documented in Florida (Tiwari et al. 2011a, Kanga et al. 2016); therefore, a deeper understanding of soil-applied neonicotinoids was warranted for development of future resistance management strategies. We observed a number of effects that are of significant concern regarding use of neonicotinoids by soil application in Florida citrus: i) failure to achieve lethal concentrations in leaf tissue following application to the soil; ii) persistence of thiamethoxam concentrations at low levels (less than 1 ppm) through 12 wk following application; and iii) failure of the highest allowable annual rate to achieve acceptable *D. citri* control following application to trees 18 mo of age (MCV = 1.34 m^3^). Resistance management is critical for neonicotinoid stewardship to ensure implementation of the chemical class in future citrus production. Therefore, the current results suggest that application of neonicotinoids to the soil, particularly in trees with a developed canopy (those greater than 18 mo), may result in neonicotinoid titers that cause selection pressure for evolution of resistance in current populations of *D. citri* in FL. The significant reduction in titer between small trees and large trees after soil application may simply be a result of dilution of chemical due to an increase in canopy size, application method in relation to root distribution under the canopy, or perhaps due to compromise of the root system caused by *C*Las infection resulting in reduced uptake of available compound. If the latter is true, soil-applied neonicotinoids may work better when applied to trees not compromised by HLB. Because the trees used in this study were insecticide-free prior to each application, it is likely that all trees, particularly of the large size, had some level of *C*Las infection, which may have negatively influenced uptake efficiency. As an alternative to soil-applied neonicotinoids, subsequent investigations should evaluate foliar neonicotinoid applications for management of nonbearing citrus and resulting coverage uniformity, peak residue levels, degradation over time, and efficacy.

Follow-up comparative investigations quantifying the concentration of thiamethoxam, imidacloprid, and clothianidin required to interrupt and manipulate feeding behavior of *D. citri* that utilize electropenetrography are warranted to further improve the use of these management tools for *D. citri* and HLB in citrus. Moreover, alternative soil application methods should be investigated that attempt to increase uptake efficiency, particularly in trees 18 mo and older. Given the dynamic nature of susceptibility of *D. citri* to insecticides, we must remain diligent in research efforts with a keen focus on resistance management and be willing to adjust insecticide use patterns to ensure the longevity of each available chemical class.

## Supplementary Data

Supplementary data are available at *Journal of Economic Entomology* online.

LangdonSchumannStelinskiRogers_Matrix_Suppl_Tab_S1

LangdonSchumannStelinskiRogers_Matrix_Suppl_Tab_S2
