# Supplementary material for: Influence of Tree Size and Application Rate on Expression of Thiamethoxam in Citrus and Its Efficacy Against Diaphorina citri (Hemiptera: Liviidae)
Source: J Econ Entomol. 2018 Feb 20;111(2):770–9. doi: 10.1093/jee/toy001 (PMC6019049; doi:10.1093/jee/toy001)
Supplement: LangdonSchumannStelinskiRogers_Matrix_Suppl_Tab_S1 [file toy001_suppl_langdonschumannstelinskirogers_matrix_suppl_tab_s1.docx]

**Supplemental Table S1: Probability of encountering a *Diaphorina citri* adult on young citrus trees based on thiamethoxam titer in leaf tissue.**

| **Probability** | **Concentration (ppm)** | **95% Fiducial Limits** | |  | **Probability** | **Concentration (ppm)** | **95% Fiducial Limits** | |
| --- | --- | --- | --- | --- | --- | --- | --- | --- |
| **0.01** | 64.62813 | 34.40416 | 147.16423 |  | **0.55** | 0.24763 | 0.18915 | 0.31486 |
| **0.02** | 34.81426 | 19.88874 | 72.07353 |  | **0.60** | 0.18534 | 0.13803 | 0.23892 |
| **0.03** | 23.51246 | 14.03654 | 45.85806 |  | **0.65** | 0.13737 | 0.09920 | 0.18047 |
| **0.04** | 17.50123 | 10.79389 | 32.65360 |  | **0.70** | 0.10019 | 0.06976 | 0.13481 |
| **0.05** | 13.76460 | 8.71368 | 24.78236 |  | **0.75** | 0.07127 | 0.04754 | 0.09874 |
| **0.06** | 11.21976 | 7.25962 | 19.60355 |  | **0.80** | 0.04877 | 0.03093 | 0.07002 |
| **0.07** | 9.37867 | 6.18388 | 15.96622 |  | **0.85** | 0.03135 | 0.01868 | 0.04705 |
| **0.08** | 7.98808 | 5.35517 | 13.28972 |  | **0.90** | 0.01797 | 0.00988 | 0.02861 |
| **0.09** | 6.90326 | 4.69711 | 11.25013 |  | **0.91** | 0.01571 | 0.00846 | 0.02538 |
| **0.10** | 6.03539 | 4.16204 | 9.65293 |  | **0.92** | 0.01358 | 0.00716 | 0.02229 |
| **0.15** | 3.46039 | 2.51446 | 5.13735 |  | **0.93** | 0.01157 | 0.00595 | 0.01932 |
| **0.20** | 2.22395 | 1.67583 | 3.12827 |  | **0.94** | 0.00967 | 0.00484 | 0.01648 |
| **0.25** | 1.52197 | 1.17661 | 2.05542 |  | **0.95** | 0.00788 | 0.00382 | 0.01374 |
| **0.30** | 1.08264 | 0.85119 | 1.41831 |  | **0.96** | 0.00620 | 0.00290 | 0.01111 |
| **0.35** | 0.78960 | 0.62631 | 1.01252 |  | **0.97** | 0.00461 | 0.00206 | 0.00855 |
| **0.40** | 0.58525 | 0.46475 | 0.74073 |  | **0.98** | 0.00312 | 0.00131 | 0.00604 |
| **0.45** | 0.43802 | 0.34567 | 0.55145 |  | **0.99** | 0.00168 | 0.0006408 | 0.00350 |
| **0.50** | 0.32934 | 0.25652 | 0.41537 |  |  | | | |
