# Supplementary material for: Influence of Tree Size and Application Rate on Expression of Thiamethoxam in Citrus and Its Efficacy Against Diaphorina citri (Hemiptera: Liviidae)
Source: J Econ Entomol. 2018 Feb 20;111(2):770–9. doi: 10.1093/jee/toy001 (PMC6019049; doi:10.1093/jee/toy001)
Supplement: LangdonSchumannStelinskiRogers_Matrix_Suppl_Tab_S2 [file toy001_suppl_langdonschumannstelinskirogers_matrix_suppl_tab_s2.docx]

**Supplemental Table S2: Probability of encountering a *Diaphorina citri* nymph on young citrus trees based on thiamethoxam titer in leaf tissue.**

| **Probability** | **Concentration (ppm)** | **95% Fiducial Limits** | |  | **Probability** | **Concentration (ppm)** | **95% Fiducial Limits** | |
| --- | --- | --- | --- | --- | --- | --- | --- | --- |
| **0.01** | 19.05254 | 12.16790 | 34.07621 |  | **0.55** | 0.59899 | 0.49336 | 0.72459 |
| **0.02** | 12.96913 | 8.64334 | 21.89294 |  | **0.60** | 0.50024 | 0.40916 | 0.60505 |
| **0.03** | 10.16081 | 6.95350 | 16.54363 |  | **0.65** | 0.41525 | 0.33608 | 0.50386 |
| **0.04** | 8.45671 | 5.90171 | 13.40459 |  | **0.70** | 0.34126 | 0.27223 | 0.41687 |
| **0.05** | 7.28366 | 5.16330 | 11.29901 |  | **0.75** | 0.27613 | 0.21615 | 0.34088 |
| **0.06** | 6.41433 | 4.60705 | 9.77162 |  | **0.80** | 0.21812 | 0.16664 | 0.27335 |
| **0.07** | 5.73789 | 4.16809 | 8.60486 |  | **0.85** | 0.16570 | 0.12263 | 0.21204 |
| **0.08** | 5.19299 | 3.81004 | 7.68013 |  | **0.90** | 0.11725 | 0.08306 | 0.15463 |
| **0.09** | 4.74248 | 3.51067 | 6.92674 |  | **0.91** | 0.10786 | 0.07556 | 0.14335 |
| **0.10** | 4.36242 | 3.25549 | 6.29960 |  | **0.92** | 0.09850 | 0.06817 | 0.13204 |
| **0.15** | 3.08692 | 2.37788 | 4.25992 |  | **0.93** | 0.08914 | 0.06086 | 0.12066 |
| **0.20** | 2.34503 | 1.84788 | 3.12933 |  | **0.94** | 0.07974 | 0.05361 | 0.10913 |
| **0.25** | 1.85240 | 1.48480 | 2.40760 |  | **0.95** | 0.07023 | 0.04638 | 0.09735 |
| **0.30** | 1.49887 | 1.21695 | 1.90723 |  | **0.96** | 0.06048 | 0.03911 | 0.08514 |
| **0.35** | 1.23180 | 1.00945 | 1.54090 |  | **0.97** | 0.05034 | 0.03170 | 0.07224 |
| **0.40** | 1.02251 | 0.84303 | 1.26208 |  | **0.98** | 0.03944 | 0.02396 | 0.05809 |
| **0.45** | 0.85394 | 0.70607 | 1.04352 |  | **0.99** | 0.02685 | 0.01540 | 0.04125 |
| **0.50** | 0.71519 | 0.59118 | 0.86814 |  |  | | | |
